# Predicting rarity and decline in animals, plants, and mushrooms based on species attributes and indicator groups

**DOI:** 10.1002/ece3.699

**Published:** 2013-08-28

**Authors:** C J M Musters, Vincent Kalkman, Arco van Strien

**Affiliations:** 1 Institute of Environmental Sciences, Leiden UniversityP.O. Box 9518, 2300, RA Leiden, The Netherlands; 2 European Invertebrate Survey – the Netherlands, Naturalis Biodiversity CenterP.O. Box 9517, 2300 RA, Leiden, The Netherlands; 3 Statistics NetherlandsP.O. Box 24500, 2490, HA The Hague, The Netherlands; 4 Institute for Biodiversity and Ecosystem Dynamics, University of AmsterdamScience Park 904, 1098, XH Amsterdam, The Netherlands

**Keywords:** Birds, butterflies, random forests, Red Lists, vascular plants, vertebrates

## Abstract

In decisions on nature conservation measures, we depend largely on knowledge of the relationship between threats and environmental factors for a very limited number of species groups, with relevant environmental factors often being deduced from the relationship between threat and species traits. But can relationships between traits and levels of threats be identified across species from completely different taxonomic groups; and how accurately do well-known taxonomic groups indicate levels of threat in other species groups? To answer these questions, we first made a list of 152 species attributes of morphological and demographic traits and habitat requirements. Based on these attributes we then grew random forests of decision trees for 1183 species in the 18 different taxonomic groups for which we had Red Lists available in the Netherlands, using these to classify animals, plants, and mushrooms according to their rarity and decline. Finally, we grew random forests for four species groups often used as indicator groups to study how well the relationship between attribute and decline within these groups reflected that relationship within the larger taxonomic group to which these groups belong. Correct classification of rarity based on all attributes was as high as 88% in animals, 85% in plants, and 94% in mushrooms and correct classification of decline was 78% in animals, 69% in plants, and 70% in mushrooms. Vertebrates indicated decline in all animals well, as did birds for all vertebrates and vascular plants for all plants. However, butterflies poorly indicated decline in all insects. Random forests are a useful tool to relate rarity and decline to species attributes thereby making it possible to generalize rarity and decline to a wider set of species groups. Random forests can be used to estimate the level of threat to complete faunas and floras of countries or regions. In regions like the Netherlands, conservation policy based on attributes known to be relevant for the decline to birds, vertebrates or plants will probably also impact all aboveground terrestrial and freshwater macrofauna or macrophytes.

## Introduction

Many countries have ratified the Convention of Biological Diversity, thereby agreeing to protect their biodiversity and to prevent extinction of their native species (http://www.cbd.int/convention/parties/list/). Globally, the number of known species is estimated to be around 1.24 million, with many more species as yet unknown (Mora et al. 2011). This vast number of species makes it difficult to inform policy-makers and the general public on changes in biodiversity because it is virtually impossible to monitor all species. As a result, it is difficult to ascertain which species are in most urgent need of conservation measures, and whether conservation measures are sufficient to prevent extinctions.

Out of sheer necessity, policy-makers and nature managers generally focus on a limited number of selected species, thereby assuming that these are representative of all native species. The European Union, for instance, focuses on the protection of birds through the Bird Directive and on a scatter of other species through the Habitat Directive (http://bd.eionet.europa.eu/activities/Natura_2000/reference_portal). In this selection, vertebrates and butterflies are clearly overrepresented (Henle et al. 2013). In global assessments of biodiversity change, vertebrates are also overrepresented (Millennium Ecosystem Assessment 2005; Butchart et al. 2010). For example, the most widely used indicator of global biodiversity change, the Living Planet Index, is an aggregated statistic composed of trends in vertebrate species only, in which birds and mammals are currently overrepresented (Loh et al. 2005).

Although there may be good reason to focus on vertebrates and other more visible species, such as butterflies, rather than selecting species hardly recognized by the general public, a key question remains whether the species selected for use in nature policy and management may be regarded as representing all species (Thomas et al. 2004). In an effort to test the issue of representativeness, some authors have examined whether trends in one species group were similar to those in other species groups (Thomas et al. 2004). Others have compared the overlap of diversity hotspots between species groups (Reid 1998; Heino 2002; Fontaine et al. 2007). If trends or hotspots coincided, one could thin out the number of species groups that need to be taken into account for information on the need and progress in conservation actions. However, even if trends and hotspots coincided across species groups – and often they do not – it remains unclear whether the environmental factors determining trends and hotspots are the same. Consequently, generalization of findings beyond the species groups studied is difficult.

A more fruitful approach often applied is to examine which traits make species vulnerable to threats because traits may be linked to the environmental factors causing the species to decline (Webb et al. 2010). The advantage of this approach is that it makes it easier to generalize findings beyond the particular species studied. Most studies on the relationship between traits and threat status are within-species group studies, for example, in birds (Julliard et al. 2003; Jiguet et al. 2007; van Turnhout et al. 2010; Végvári et al. 2010), fowl (Keane et al. 2005), bats (Jones et al. 2003), butterflies (Kotiaho et al. 2005), moths (Mattila et al. 2006), and beetles (Davies et al. 2004). Several other studies have involved cross-taxon analyses, mainly to assess common or different traits across a handful of species groups (mammals: Purvis et al. 2000; four groups of invertebrates: Kleukers and Reemer 2003; three groups of vertebrates: Collen et al. 2006; mammals and arthropods: Jennings and Pocock 2009; tropical forest species: Stork et al. 2009; three invertebrate groups and birds: Vandewalle et al. 2010).

Here, we aim to find a method that is universal in the sense that it predicts whether a species is rare or in decline across multiple taxonomic groups based solely on traits and other species attributes. This would enable us to estimate the level of threat of complete faunas and floras of countries and regions. To do so, we examined the relationship between rarity and decline in 1183 Dutch species of 18 different taxonomic groups and 61 species traits, transformed into 152 attributes. We use a broad definition of “trait”, including morphological and demographic traits as well as habitat requirements of species.

To study the relevance of traits for threats, regression analysis is a favored approach (Purvis et al. 2000; Julliard et al. 2003; Hero et al. 2005; Keane et al. 2005; Fréville et al. 2007; Sodhi et al. 2008; van Turnhout et al. 2010). However, regression analysis has considerable drawbacks, in particular because it is difficult to treat many different traits, to include nonadditive and nonlinear relationships between trends and traits, and to handle nonadditivity, nonlinearity, collinearity, and interactions between traits (Bielby et al. 2010). Decision trees are an alternative method (Jones et al. 2006; Sullivan et al. 2006; Olden et al. 2008; Davidson et al. 2009; Bielby et al. 2010). These may perform better in categorizing species than regression-based approaches, as they suffer less from all the aforementioned difficulties (Jones et al. 2006). They treat nonlinearity and interactions without the need to incorporate these features explicitly a priori in a model (Fréville et al. 2007). Besides, there is no a priori need for trait selection in the case of a high number of traits or collinearity. Another advance over regression approaches is that pseudoreplication due to phylogenetic relationships between species is no longer an issue (Jones et al. 2006; Bielby et al. 2010; Murray et al. 2011).

A recent development in decision tree analysis is the use of random forests, a well-established technique in machine learning but relatively new to ecology (Breiman 2001; Cutler et al. 2007; Boyer 2009; Davidson et al. 2009; Murray et al. 2011). Random forests consist of a large number of decision trees, each based on a random sample of species and traits to prevent overfitting (Breiman 2001). They have the advantage that there is no need to omit part of the data set from the training data set for use in validation because each decision tree of the forest is grown based on a subset of the species, with the classification error of the tree being monitored on the other species (out-of-bag approach, Breiman 2001).

Our first main research question is focused on the performance of random forests: How well do random forests of decision trees grown from data of taxonomically very different species predict rarity and decline in the species? For our second main question we use random forests to get insight in the indicative value of specific taxonomic groups: What is the indicative value of vertebrates, birds, butterflies and vascular plants for all animals, vertebrates, insects, and all plants, respectively?

Rarity and decline in the studied species were derived from the existing Red Lists of the Netherlands. The Dutch species groups evaluated for the Red Lists are not a representative sample of all the species groups occurring on Dutch territory (Noordijk et al. 2010). A preliminary comparison of the species evaluated for Red Lists and all known Dutch species showed that small species, marine species, and soil species are underrepresented. Therefore, our data set could be regarded as representative for aboveground terrestrial and freshwater macrofauna, macrophytes, and macrofungi. We believe our results can be regarded as indicative for areas like the Netherlands, that is, temperate areas with a high degree of urbanization and intensive land use.

## Material and Methods

### Selection of species

In the Netherlands, Red Lists are available for 18 taxonomic groups (Table S1). All Dutch species within these groups are evaluated for the Red Lists, except those for which rarity or decline is insufficiently known. A total of 6097 evaluated species are available from which 1183 species were selected for further analysis (Table S1). A random selection from all evaluates species would yield virtually only plant and fungus species. To achieve better distribution across the taxonomic groups, we randomly selected a number of species within each group. This number was proportional to the natural logarithm of the number of species per group or the number based on equal numbers of species per group, whichever was higher. In addition, from groups with only a few species, for example, reptiles, we selected all species.

As no validation data set is needed when applying Random Forest analyses, all the latter species could be included in our analyses. Because for some species the experts were not able to find all information needed, the actual number of species for analyses were 622 animals, 222 plants, and 248 mushrooms.

According to the Dutch Red List criteria, a species' threat status is determined by the trend since 1950 and its current range or abundance within the Netherlands. Rarity and decline categories of the Dutch Red Lists are based on information on past and present distribution, corrected for known biases due to differences in research effort between species, but may differ slightly among species groups. They are generally accepted by experts as the best estimates of the actual rarity and decline in the species. Analyses were performed on rarity and decline, and not on Red List status because these two criteria are the ecological features of a species that might be causally related to traits. “Rarity” was defined as a binary that states whether the species is rare in the Netherlands (“rare” and higher categories in the Dutch Red Lists), “decline” as a binary indicating whether the species range is declining in the Netherlands (“moderately declining” and higher categories in the Dutch Red Lists) (for details see de Iongh and Bal 2007). These definitions were chosen so that prevalence of rarity and decline, that is, the number of rare and declining species divided by the total number of species within our data set, was as close as possible to 0.5, as random forests perform best when class membership is approximately equal (Murray et al. 2011). Overall prevalence was 0.57 for rarity and 0.46 for decline.

In nature conservation policy-making and management, groups that are often implicitly regarded as indicator groups are the vertebrates (e.g., Loh et al. 2005), birds (e.g., Gregory et al. 2009), butterflies (e.g., Thomas 2005), and vascular plants (Vamosi and Vamosi 2008). For studying the indicative value of these groups for the higher taxonomic group to which they belong, we grew random forests for the decline in our sample of the species in the indicator group. We then used these random forests to classify our sample of the higher taxonomic group into either decreasing or nondecreasing species. In the case of the vertebrates, the higher taxonomic group included all animal species; with the birds, all vertebrates; with the butterflies, all insects; and with the vascular plants, all plants.

### Selection of traits

To identify traits predicting rarity and decline in species across taxonomic groups, we had to find traits that are shared by as many species as possible and that are ecologically relevant.

We distinguished four main categories of traits that are known to be relevant for the range or abundance of a species (rarity) and its change in range or abundance (decline). The first category is formed by traits related to the niche of the species; these traits are connected to abiotic or biotic factors or susceptibility to isolation (Pulliam 2000; Silvertown 2004; Soberón 2007). Traits connected to abiotic factors include habitat and climatic requirements. Traits connected to biotic factors include trophic level and competitive strength. Among the factors reinforcing isolation are poor dispersion capacity or occurrence in isolated habitat types. The second category contains traits related to direct human influence. Species may, for example, be harvested or protected by humans. Certain traits may make species more vulnerable to stochastic processes than others. Traits related to stochastic processes are therefore regarded as a third category. Stochastic processes are usually subdivided into genetic, population-dynamic, and environmental stochastic processes. Examples include the number of eggs per female and life span. While the first three categories treat traits as fixed characteristics, the fourth category contains traits related to the flexibility of traits, that is, trait evolvability and trait plasticity (Forsman and Hagman 2009).

All four categories were further subdivided, leading to a list of 61 traits intended to be as complete as possible (Table 1). To examine whether important traits may have been missed, we compared our list with traits cited in the literature. Traits marked with an X in Table 1 were also indicated as relevant in at least one of 32 recent articles (Supporting Information). Certain traits found in the literature could not easily be included in our categorization. These traits appeared to be either species group specific (e.g., nest site in birds) or covered by a combination of traits in our list (e.g., habitat disturbance in plants).

**Table 1:** Traits of importance for species rarity and decline

Category		Subcategory	Trait	R	Attributes
Niche	Abiotic	Habitat	Marine: rich vs. poor soil structure		15,35
			Aquatic: stagnant vs. running	X	15,18
			Land cover: open vs. closed	X	15,16,50
		Climate	Global range: northern, southern, eastern, western	X	
			Altitude: lowland vs. mountain species; sea depth	X	
			Seasonality:		
			Plants: deciduous or evergreen		55
			Animals: migrating, hibernating	X	38
			Breeding/flowering/flying period	X	22
			Day/night active	X	39
		Energy	Body mass	X	
			Length (at maturity)	X	10
			Length-body mass ratio	X	
			Animals: Ecto- vs. endotherm		37
			Plants: preference for shaded or nonshaded habitats		50
		Macronutrients and water	High vs. low productive habitat	X	57,61,62,65
			Dry, humid, aquatic habitat		17,56
			Size of home range	X	36
			Endo- or exoskeleton containing chalk		
		Respiration	Oxygen-rich vs. -poor habitat		
		Vulnerability for toxins etc.	Vulnerably due to intake nutrients and water via skin and gut	X	
			Vulnerably due to intake due to respiration via skin, gills, lungs	X	
	Biotic	Food	Photosynthesis, detrivore, parasite, predator, herbivore, omnivore	X	23,24,40
		Food web	Number of species:	X	41,42,43
			On which species depend for food		
			That depend on species as food source		
		Competition	Intraspecific:		
			Animals: solitary vs. social (group size)	X	44
			Plants: life form (Raunkiaer system)	X	51,63,64
			Interspecific: number of species in the same guild		
		Mutualism/symbioses	Number of species on which species depend for living space, reproduction, pollination, transport, etc.	X	34,47,52
	Accessibility	Dispersion	Animals: immobile, walking, flying, swimming, carried	X	45,46
			Plants and fungi propagule dispersion through soil, water, wind, animals	X	53
			Dispersal distance		13,14,25
		Isolation habitat	Habitat common vs. rare	X	56,57
			Habitat difference from matrix: weak vs. strong (ponds, islands, mountain tops, etc.)	X	
Human		Food/material	Species collected or harvested	X	4
		Biophilia	High vs. low appreciation of species		
		Predators/parasites	Species dangerous or considered a pest		26
		Management	Species protected, managed or controlled	X	3
		Mutualism/symbioses	Species dependent on urban or agricultural areas	X	20,27,28
			Dispersal by humans: invasive species		6
Stochastic	Genetic	Effective population size	Local population size/density	X	59
		Genetic diversity	Global population size	X	
			Known bottleneck	X	
	Population dynamics	Population stability	Fecundity (number of propagules per female per year)	X	48
			Egg/propagule weight	X	
			Number of generations per year	X	29
			Distinct gametophyte/larva stage	X	30
			Distinct male/female dimorphism	X	
			Development time/age at maturity	X	31
			Life span/max. age	X	32
			Parental care	X	
	Environmental	Habitat stability	Habitat stability		19,21
			Distinct habitat of gametophyte/larva	X	49
			Propagation strategy: nonsexual vs. sexual	X	33
			Resting stages:		
			Plants: seed longevity	X	54
			Animals: month/years in diapauses, etc.	X	
		Disasters			
	Flexibility of traits	Delimiting: bauplan			
		Evolvability	Number of species within same genus [family]	X	1
			Number of subspecies within same species	X	2
			Number of morphs/varieties/aberrations within same species		
		Plasticity	Number of growth forms within same species		

Column R indicates analysis of the trait in one or more references, the last column which attributes were used in this study as a proxy for the trait. Attribute numbers are specified in Table S2.

The traits listed in Table 2 were used to design a questionnaire that was sent to species group experts, who were asked to fill in the traits for the species within “their” species group. Apart from the answers to these questionnaires, for most of the species distribution within the Netherlands was also known, both in the past and present (e.g., Hustings and Vergeer 2002; Creemers and van Delft 2009). This information was used to assess the range of the species in the Netherlands between 1950 and 1990. It was also used to assess the species' preference for certain land-use categories (LUC) and physical–geographical regions (PGR).

**Table 2:** Classification of rarity and decline by attributes

	Species (*n*)	Attributes available of	Classification			Error probability	
		
			Attributes (*n*)	Correct (%)	*P*-value chi-square	Type I	Type II
Rarity							
Animals	622	Evaluated sp.	129	87.94	<0.001***	0.109	0.131
		Well-known sp.	88	67.52	<0.001***	0.321	0.328
		Poorly known sp.	40	66.56	<0.001***	0.369	0.304
Plants	222	Evaluated sp.	75	84.68	<0.001***	0.218	0.099
		Well-known sp.	44	73.87	<0.001***	0.317	0.215
		Poorly known sp.	31	72.97	<0.001***	0.356	0.198
Mushrooms	248	Evaluated sp.	64	94.35	<0.001***	0.088	0.042
		Well-known sp.	30	71.77	<0.001***	0.488	0.185
		Poorly known sp.	17	65.32	0.239 NS	0.825	0.119
Decline							
Animals	622	Evaluated sp.	130	76.85	<0.001***	0.159	0.312
		Well-known sp.	88	69.45	<0.001***	0.275	0.340
		Poorly known sp.	40	64.31	<0.001***	0.336	0.380
Plants	222	Evaluated sp.	76	68.92	<0.001***	0.258	0.383
		Well-known sp.	44	61.26	0.004**	0.297	0.511
		Poorly known sp.	31	61.26	0.003**	0.305	0.500
Mushrooms	248	Evaluated sp.	65	70.16	<0.001***	0.259	0.345
		Well-known sp.	30	59.27	0.004**	0.385	0.434
		Poorly known sp.	17	45.97	0.182 NS	0.519	0.566

The answers to the questionnaires, together with information on distribution of the species, were our independent variables. In accordance with decision tree literature, we called these variables the attributes of the species. In several cases more than one attribute could be regarded as reflecting a certain trait. Some traits turned out to be irrelevant for the Dutch species (e.g., altitude). In some cases, we did not succeed to collect information on the trait (e.g., intake of oxygen or nutrients thru skin) or assumed that the trait was correlated with other traits (e.g., body mass and body length) (Table 1).

All attributes were transformed into categorical variables in order to avoid the influence of cardinality on the importance of attributes (Deng et al. 2011). In the case of existing categorical variables of no relevance for certain species (whether a species prefers stagnant or running water is of no relevance for nonaquatic species), the variable was transformed into a dummy attribute (species of stagnant water; species of running water) so that the species for which the variable had no relevance had zeros for all the dummy attributes. In the case of scale variables, the scale was divided into five equal parts, leading to a five-point ordinal attribute. When needed, the raw values of the scale were log transformed for approaching a normal distribution before this transformation into an ordinal attribute. For preferences of species for a LUC or a PGR the group-equalized phi-coefficient was calculated (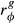, De Cáceres and Legendre 2009). LUC and PGR “specialization” is the square root of the sum of squares of the phi-coefficients of, respectively, all LUC and PGR categories. “Commonness 1950–1990” is the logit transformation of the number of all Dutch grid cells in which the species was observed at least once over the complete period divided by the number of grid cells in which the species group was observed in the period 1950–1990. This attribute was not included in the analyses of rarity. The maximum number of categories of an attribute is 10, the minimum number two, but most attributes have either two or five categories (Table S2).

Attributes that turned out to have more than 99% of the species in one category were omitted from the analyses because these attributes were deemed to be uninformative. Attributes that had more than 25% values missing were also omitted because it was feared that replacing missing values by imputed values might introduce a bias in attributes with a large number of missing values.

### Attribute availability

All the species in our analyses have been evaluated for Red List status and are therefore well studied. However, if the results of our analyses are to be used to estimate the threat to all the species of the Netherlands, one of our ultimate goals, due consideration should be given to the fact that for most of the nonevaluated species much less information is available on traits and distribution. To be able to study the effect of this possible lack of information on species classification, we drew up three groups of attributes based on the expected availability of information. All our attributes are attributes known for the “*evaluated species”*. Of these, a subset is known for the *“well-known species”*. These attributes cover ecological and behavioral information, but not distribution. The attributes known for the *“poorly-known species”* are again a subset of these attributes. They include morphological and taxonomical information. This classification of attributes was based on expert judgment and can be found in our overview of attributes in Table S2.

### Random forests

We applied Random Forest analysis, using the package “randomForest” of R 2.12.2 (version 4.6-6; Breiman and Cutler 2012). The random forests were evaluated by means of correctness of classification, that is, the proportion of species correctly classified. We tested whether the rare or declining species were classified differently from not rare or not declining species or whether two classifications differed significantly from each other with the Pearson Chi-square test of R. For more insight, we also give the risks of classification errors, that is, the *false*-*positive rate* or *probability of type I errors*: the probability of a species that is common or not declining being classified as rare or declining, and the *false*-*negative rate* or *probability of type II errors*: the probability of a species that is rare or declining being classified as common or not declining (Fig. S1). The probability of Type I errors should be low in order to minimize the risk of limited resources for conservation policy being used for nonthreatened species, whereas the probability of Type II errors should be low to minimize the risk of a threatened species not being recognized as such. For proper classification, then, both Type I and Type II error probabilities should be low. In our discussion, we regard probabilities of errors less than 0.2 as “low”.

For each random forest grown we followed the same procedure. First, the missing values in the data set were replaced by imputed values, which were based on the values of proximate species according to 1000 decisions trees. Imputing was iterated 10 times. Then, a random forest of 10,000 trees was grown from the imputed data set. The defaults of the randomForest package were kept for the number of species that were randomly selected for each tree, as for the number of attributes that were randomly tested at each node as candidates for the split (Breiman and Cutler 2012). Stability of the error classification was always checked visually. In case of the analyses of the indicative value of specific species groups, the influence of the random parts of the procedure on the outcome was checked by growing the random forest, including the imputation, 10 times. In the results, these analyses can be recognized by the error bars.

The importance of an attributes for the classification of the species can be estimated by comparing the correct classification of the random forest with that of a random forest in which the values of the attributed are randomly permuted (Breiman and Cutler 2012). The larger the decrease in correct classification, the more important the attribute is. As we have no formal way of making a distinction between the “really” important attributes and the others, we arbitrarily give only the 10 most important attributes in order of importance in the results.

When classifying the species of a higher taxonomic group using a random forest of an indicator group, we only classified the decline in species, assuming that it is most relevant for conservation. We used the imputed data of these species because we did not want the evaluation of the indicative value of the indicator group to be affected by an unbalanced lack of information.

## Results

### Classification of animals, plants, and mushrooms by attributes

When random forests were grown based on all available attributes in our data set, these classified 87.9% of the animals, 84.7% of the plants, and 94.3% of the mushrooms to the correct rarity class (Table 2). The probabilities of Type I and Type II errors were small in all cases, except for the Type I error in plants, which was over 0.2. In all three random forests almost all important attributes were preferences for certain LUC and PGR (Table 3).

**Table 3:** The ten most important attributes per random forest of rarity and decline in order of importance

Attributes available of	Animals	Plants	Mushrooms
Rarity			
Evaluated species	PGR Open water	PGR Open water	LUC Open water
	LUC Estuarine marshland and tidal sand plates	PGR Specialization	PGR Open water
	LUC Arable land	LUC Open water	LUC Estuarine marshland and tidal sand plates
	LUC Specialization	PGR Marine clay	LUC Orchards
	PGR Anthropogenic	LUC Specialization	LUC Specialization
	Recent range largely in agric. area	Functional group	LUC Grassland
	LUC Grassland	LUC Urban area	PGR Specialization
	PGR Specialization	PGR Peat	LUC Arable land
	PGR River clay	Natural habitats	PGR Marine clay
	LUC Open water	LUC Deciduous woods	LUC Deciduous woods
Well-known species	Number of offspring per year	Functional group	Reproductive period July–September
	Number of species of genus	Body size	Reproductive period October-March
	Western border of range through NL	Number of species of genus	Functional group
	Active dispersion	Dispersion capacity	Number of species of genus
	Dispersion capacity	Seed longevity >10 years	Taxonomic group
	Number of subspecies of species	Northern border of range through NL	Body size
	Running aquatic habitats	Reproductive period July–September	Mainly nonforested habitats
	Northern border of range through NL	Seed longevity <4 years	Forested and nonforested habitats
	Number of generations per year	Year before reproduction	Exclusively forested habitats
	Body size	Seed longevity 4–10 years	Humid hab
Poorly known species	Number of offspring per year	Functional group	Functional group
	Number of species of genus	Body size	Number of species of genus
	Number of subspecies of species	Number of species of genus	Taxonomic group
	Running aquatic habitats	Seed longevity >10 years	Body size
	Nonadult morphologically different	Seed longevity <4 years	Shaded habitats
	Body size	Seed longevity 4–10 years	Shaded and nonshaded habitats
	Endemic	Year before reproduction	Nonshaded habitats
	Reproductive area	Harvested	Parasitic
	Reproductive years	Number of subspecies of species	Depending on symbiosis
	Running and stagnant aquatic habitats	Winter leaf carrying	Living of dead material
Decline	Commonness 1950–1990	Years before reproduction	LUC Deciduous woods
Evaluated species	PGR Open water	Commonness 1950–1990	Commonness 1950–90
	LUC Deciduous woods	Functional group	Functional group
	Natural habitats	LUC Urban area	Sensitivity eutrophication
	Recent range largely in agric. area	Number of species of genus	PGR Loess
	Active dispersion	Natural habitats	Number of species of genus
	Number of offspring per year	PGR Marine clay	PGR Open water
	LUC Arable land	Habitat stability	PGR Old clay
	PGR Anthropogenic	PGR Specialization	Taxonomic group
	LUC Estuarine marshland and tidal sand plates	LUC Specialization	Habitat stability
Well-known species	Number of offspring per year	Functional group	Functional group
	Number of species of genus	Years before reproduction	Number of species of genus
	Active dispersion	Number of species of genus	Taxonomic group
	Dispersion capacity	Body size	Body size
	Number of subspecies of species	Dispersion capacity	Reproductive period July–September
	Number of generations per year	Northern border of range through NL	Reproductive period October-March
	Body size	Seed longevity <4 years	Forested and nonforested habitats
	Western border of range through NL	Endemic	Exclusively forested habitats
	Reproductive area	Seed longevity >10 years	Dry habitats
	Mainly nonforested habitats	Reproductive years	Humid habitats
Poorly known species	Number of offspring per year	Functional group	Functional group
	Number of species of genus	Years before reproduction	Number of species of genus
	Number of subspecies of species	Number of species of genus	Taxonomic group
	Body size	Body size	Body size
	Nonadult morphologically different	Seed longevity <4 year	Shaded habitats
	Running aquatic habitats	Seed longevity >10 years	Shaded and nonshaded habitats
	Year before reproduction	Reproductive years	Nonshaded habitats
	Endemic	Seed longevity 4–10 years	Parasitic
	Reproductive area	Endemic	Photosynthetic
	Running or stagnant aquatic habitats	Number of subspecies of species	Living of dead material

Definitions of attributes in Table S2; LUC, land-use category; PGR, physical–geographical region; NL, Netherlands.

**Figure 1:**
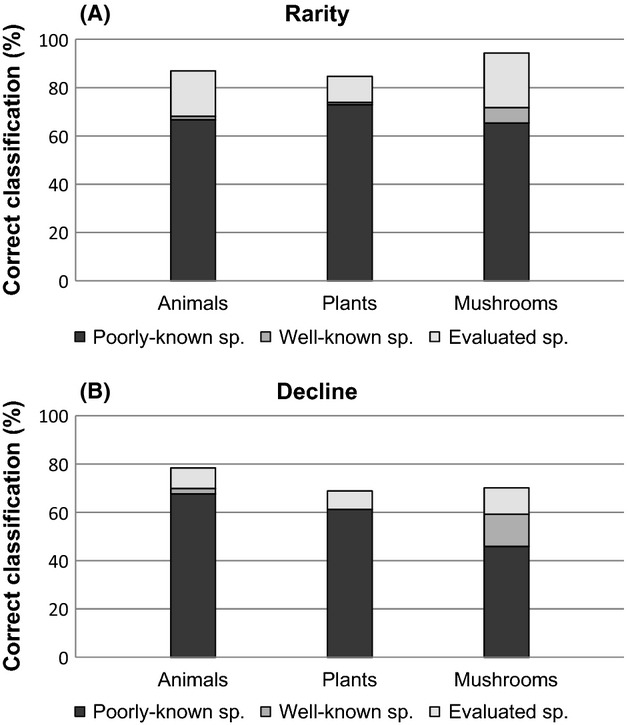
Improvement of correct classification of (A) species rarity and (B) species decline by random forests going from using attributes available of poorly known species to those of well-known species and of species evaluated for Red Lists.

If these random forests were to be used to classify species not included in our learning set, not all attributes would be known for all species. The random forests correctly classify the rarity of 66.6% of the animals when based on attributes expected to be known for poorly known species, 73.0% of the plants, and 65.3% of the mushrooms (Table 2). In mushrooms, this classification does not differ from a random classification (Chi-square test, *P* = 0.2239; Table 2). With the lower rate of correct classifications due to fewer attributes being available, the risk of error obviously increases (Table 2). In all three cases, Type I errors have a higher probability than Type II errors. Attributes of well-known species do not significantly improve correct classification, but the improvement from attributes of well-known species to those of evaluated species is significant in all three groups (Chi-square test animals: *P* < 0.001; plants: *P* = 0.005; mushrooms: *P* < 0.001; Fig. 1).

Random forests grown based on all available attributes, correctly classified the decline in 76.9% of the animals, 68.9% of the plants, and 70.2% of the mushrooms (Table 2). Only for animals is the probability of a Type I error below 0.2. In all three cases the probability of a Type II error is higher than that of a Type I error and over 0.3. The attribute “Commonness in 1950–1990” is the only attribute that was important in animals, plants as well as mushrooms (Table 3).

Classification of decline, when based solely on the attributes of poorly known species, is correct in 64.3% of the animals, 61.3% of the plants, and 46.0% of the mushrooms. Again, in mushrooms this classification does not differ from a random classification (Chi-square test, *P* = 0.182; Table 2). In all three cases error probabilities are high, with Type II errors more probable than Type I errors (Table 2). Now, in mushrooms classification by attributes of well-known species leads to a marked improvement (Chi-square test mushrooms: *P* = 0.003). The improvement from attributes of well-known species to those of evaluated species is significant in animals and mushrooms, not in plants (Chi-square test animals: *P* = 0.003; plants: *P* = 0.091; mushrooms: *P* = 0.011; Fig. 1).

### Classification by indicator groups

The forests were able to classify the decline in 65.7% of the vertebrates species correctly, 62.3% of the birds, 87.8% of the butterflies, and 75.2% of the vascular plants (Table 4). The bird classification was no different from a random classification (Chi-square test, *P* = 0.573). In the case of vertebrates, birds, and vascular plants, the risk of Type II errors was high: around 0.5 or higher. In butterflies, the probability of Type I errors was high (0.3), but that of Type II errors extremely low (0.05). The question now is whether these generally high risks of errors affect the indicative value of the indicator groups. This was examined by applying the random forests found to the higher taxonomic group of the species concerned.

**Table 4:** Correct classification of species decline in the indicator groups

		Classification		Error probability	
			
Indicator group	Species (*n*)	Correct (%)	*P*-value Chi-square	Type I	Type II
Vertebrates	175	65.71	<0.001***	0.224	0.494
Birds	77	62.34	0.573 NS	0.176	0.769
Butterflies	49	87.76	<0.001***	0.333	0.054
Vascular plants	109	75.23	<0.001***	0.091	0.625

When the random forest of vertebrates, birds, butterflies, and vascular plants are used to classify the decline in all animals, vertebrates, insects, and plants, respectively, 73.0%, 75.4%, 57.1%, and 79.7% of the latter are correctly classified (Table 5). So, the random forests of vertebrates, birds, and vascular plants appear to classify the species in the group to be indicated better than the indicator group itself, but with the butterfly random forest that classification is much worse (compare Table 4 with Table 5). Before any conclusions are drawn from this, consideration needs to be given to two possible reasons for the difference between the classification of the indicator group and that of the group to be indicated, even in the case of exactly the same probability of Type I and Type II errors.

**Table 5:** Classification of species of higher taxonomic groups on decline by the random forests of the indicator group

					Classification			Error probability	
						
Indicator group	Prev.	Higher tax. group	Species (*n*)	Prev.	Expected correct (%)	Actual correct (%)	*P*-value chi-square	Type I	Type II
Vertebrates	0.44	Animals	622	0.47	64.79	72.99	<0.001***	0.370	0.159
Birds	0.34	Vertebrates	175	0.44	56.27	75.43	<0.001***	0.153	0.364
Butterflies	0.76	Insects	371	0.52	81.12	57.14	<0.001***	0.888	0.000
Vascular plants	0.29	Plants	222	0.42	68.29	79.73	<0.001***	0.234	0.160

The expected correct classification is the correct classification of Table 4 applied on the higher taxonomic group, that is, corrected for the difference in prevalence between the indicator group and the higher taxonomic group (see Supporting Information). Prev.: prevalence: number of declining species divided by all species.

First, prevalence in the indicator group might differ from that in the group to be indicated. The expected percentage of correct classification in Table 5 is the percentage of correct classification of the indicators (Table 4), corrected for differences in prevalence between the indicator group and the higher taxonomic group using equation (S1) (Fig. S2). Second, as the separate decision trees of a random forest are based on random selections of species and attributes, the effect of these random procedures needs to be duly considered.

**Figure 2:**
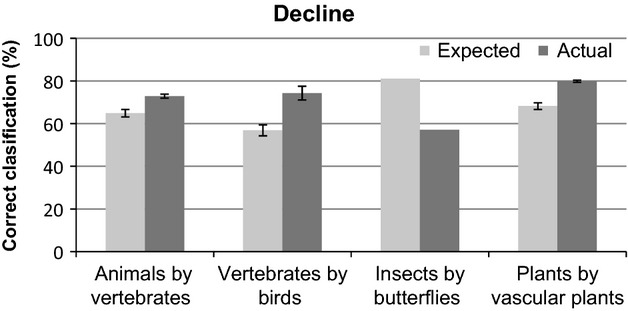
Expected classification based on correct classification within the indicator group and actual correct classification of decline in species of higher taxonomic groups by the random forests of the indicator groups. Error bars are 95% confidence intervals based on ten random forests

Figure 2 shows the difference between the expected percentage of correct classification and the actual percentage, including the effect of the random parts of the random forest procedure. The actual percentages are clearly higher in vertebrates indicating all animals, in birds indicating all vertebrates, and in vascular plants indicating all plants, but lower in butterflies indicating all insects. The fact that the butterflies perform poorly as an indicator group for insects is also obvious from the probability of errors: although this is zero for Type II errors, it is almost 0.9 for Type I errors (Table 5). Based on the probability of errors, the vascular plants perform best, with no probability of errors over 0.23. In the case of vertebrates, the risk of a Type I error is high, while that of a Type II error is low. With birds this pattern is inverted, with a low probability of a Type I error and a high probability of a Type II error (Table 5).

When indicator groups of species are used to design conservation measures intended to be effective for species outside the indicator group, too, the relationships between attribute categories and decline in the indicator group are used under the assumption that those relationships are also valid in the group to be indicated. For an initial check on this, we compared the 10 most important attributes of the random forests of the indicator group with the 10 most important attributes of the random forests of the higher taxonomic groups (Table 6). Of the 10 most important attributes of the all-animal random forest, five are also important in the vertebrate forest. The vertebrate random forest has six most important attributes in common with the bird forest. Of the 10 most important attributes of the insect random forest, only three are also important in the butterfly forest. And the all-plant random forest again has six most important attributes in common with the vascular plant forest.

**Table 6:** The ten most important attributes per random forest of decline in different species groups

Indicator groups	Higher taxonomic group
Vertebrates	Animals
Active dispersion	**Commonness 1950–1990**
Commonness 1950–1990	PGR Open water
Natural habitats	**LUC Deciduous woods**
PGR Anthropogenic	**Natural habitats**
LUC Deciduous woods	Recent range largely in agric. Area
LUC Wetlands	**Active dispersion**
LUC Urban areas	Number of offspring per year
Number of species of genus	LUC Arable land
PGR Marine clay	**PGR Anthropogenic**
Nonforest habitats	LUC Estuarine marshland and tidal sand plates
Birds	Vertebrates
Natural habitats	**Flying**
Nonforest habitats	**Commonness 1950–1990**
Number of species of genus	**Natural habitats**
PGR Peat	PGR Anthropogenic
Commonness 1950–1990	LUC Deciduous woods
Number of subspecies of species	**LUC Urban areas**
Flying	LUC Wetlands
LUC Urban areas	**Number of species of genus**
Recent range largely in agric. area	PGR Marine clay
Predator of many species	**Mainly non-forest habitats**
Butterflies	Insects
LUC Estuarine marshland and tidal sand plates	Recent range largely in agric. Area
LUC Urban greens	**FGR Open water**
Commonness 1950–1990	Number of offspring
PGR Open water	**Commonness 1950–1990**
Humidity: not wet	**LUC Estuarine marshland and tidal sand plates**
PGR Specialization	Past range largely in agric. area
LUC Open water	LUC Arable land
Winter as a nymph	LUC Fens
Number of generations per year	PGR Old clay
PGR Anthropogenic	LUC Deciduous woods
Vascular plants	Plants
LUC Urban area	**Years before reproduction**
LUC Deciduous woods	**Commonness 1950–1990**
Commonness 1950–1990	**Functional group**
PGR Specialization	**LUC Urban area**
Years before reproduction	Number of species of genus
PGR Open water	Natural habitats
PGR Marine clay	Stable habitats
PGR Anthropogenic	**PGR Specialization**
PGR River clay	**PGR Marine clay**
Functional group	LUC Specialization

Attributes in **bold** are also in the ten most important attributes in the indicator group. Definitions of attributes in Table S2; LUC, land-use category; PGR, physical–geographical region.

## Discussion

### Classification of animals, plants, and mushrooms by attributes

Random forests using traits and species from highly different groups prove to be a powerful tool for classifying species rarity when all available attributes are used. Understandably, knowledge of the preference of the species for certain LUC and PGR is a crucial factor in this high predictability of rarity. When this information is lacking, correct classification drops, with a sharp increase in the probabilities of errors.

Decline is more difficult to predict than rarity. Knowledge of preferences for LUC and PGR seems to be less vital to correct classification of decline than in the case of rarity, but knowledge of the commonness of the species in the past always contributes to the classification, which is consistent with the results of previous studies showing a negative relationship between range size and decline (e.g., Walker and Preston 2006; Murray et al. 2011).

Our results on classification of all species are better or lie in the same range as those of other studies using decision trees (Bekker and Kwak 2005; Jones et al. 2006; Olden et al. 2008; Davidson et al. 2009) or a little lower (Murray et al. 2011). However, these other studies all concerned very limited species groups with group-specific attributes and in one case including known extrinsic threats (Murray et al. 2011).

Our analyses seem to show that the amount of information used for growing a forest may be crucial. If there are only a few attributes available, as in the case of poorly known mushrooms, classification is no better than random classification. However, other studies that have applied decision tree approaches show highly correct classifications with a limited numbers of traits (Bekker and Kwak 2005; Jones et al. 2006; Olden et al. 2008; Davidson et al. 2009; Murray et al. 2011). This may be due to the fact that in these studies on specific groups, ecological knowledge was used to select specifically tailored traits. In our study, we explicitly selected nongroup-specific traits.

### Classification by indicator groups

In three of our four tests of the indicative performance of a species group, correct classifications were found to be higher than expected. In these cases, then, the random forest actually performed better on the nonlearning data set than on the learning data set. This seems counterintuitive, so how can this result been explained? It would appear that the random forests of the indicator group use attributes and decision criteria that are indeed relevant for the complete higher taxonomic groups, but that the relationship between the attributes/criteria and the decline in the higher taxonomic groups is stronger than in the indicator group, or, in other words, that the species of the higher taxonomic group show fewer exceptions to the decision rules of the random forests. This could be a consequence of the fact that the indicator group is more often the focus of human attention than the other groups, resulting in focused human activities like protection and control that may blur the relationships between ecological traits and decline. Although we endeavored to incorporate these mechanisms in our trait list, we may not have succeeded in capturing all subtleties.

Formally, it may be argued that the fact that the species of the higher taxonomic group are classified better than those of the indicator group shows that the indicative value of the indicator groups is limited. Using these indicator groups for estimating the decline in nonevaluated species will result in overestimation of the probabilities of Type I and II errors, that is, in overestimation of uncertainty. From a nature conservation point of view, however, it is actually very good news: When the attributes of these indicator random forests are used to find environmental factors for conserving species, these factors may work even better for species outside the indicator group.

Of course, our results also show that this might not always be the case. It must be concluded that butterflies are not a good indicator group for insects. Based on ecological reasoning, Thomas (2005) concluded that butterflies may be adequate indicators for terrestrial insects. Our insects included freshwater species and many other groups with ecological requirements very different from butterflies. Hence, insects are probably too heterogeneous a group to be indicated solely by butterflies.

### Applications

Our results give confidence that the random forests of all animals, plants, and mushrooms are well able to classify the aboveground terrestrial and freshwater macrofauna, macrophytes, and macrofungi according to their rarity and decline. Combining these classifications per species can then be used to estimate the Red List status of all species of which the attributes are known. This could lead to an estimation of overall level of threat of Dutch biodiversity. However, while this means that a smaller number of groups and species can be used to predict the Red List status of a much wider group, it does not render distribution data on the wider group redundant, as LUC and PGR preferences are used as attributes. Furthermore, the probability of Type I and II errors are too high to have great confidence in the prediction of the status of a single species and the method should therefore best be restricted to predicting the conservation status of groups of species. The information the model provides on the causes of threats is limited to the traits used in the model, which means that more specific questions pertaining to conservation policy cannot be addressed in any detail.

Knowing that birds, vertebrates, and vascular plants are good indicator groups for all vertebrates, all animals, and all plants, respectively, raises the question whether some of the groups now included in our data set might be redundant for getting an overall picture on levels of threat of biodiversity. Which taxonomic groups could possibly be omitted without changing the performance of our random forest significantly? A follow-up study to answer this question could result in lists of groups, or even a limited list of species, that could most efficiently deliver information on the relationship between attributes and decline in aboveground terrestrial and freshwater macrospecies. Also, nature management may be more cost-effective when resources can be devoted to a limited number of well-selected species or groups under conditions of empirically assessed assurance, with known uncertainty that other species will be protected as well.

The fact that the number of attributes may be important for finding random forests that yield good classification does not mean that attributes may not be redundant. Future studies could seek to identify those attributes that are not required for good classification. Methods for doing so are available (e.g., Cao et al. 2010).

Given the advantages of random forest techniques over regression-based techniques, cited earlier, we consider random forests to be a promising technique for studying relationships between traits and a wide variety of species characteristics relevant for policy-making. Future avenues for employing the methodology might be the study of invasiveness, pathogenicity, and range shift (Angert et al. 2011).

## Conclusions

Random forests are found to be powerful analyzing instruments. Using traits and requirements, they are able to correctly classify species in highly different taxonomic groups into categories of rarity and decline. They may therefore be helpful in finding efficient indicator sets of species and attributes.

In designing nature conservation measures we depend largely on knowledge on the relationship between threats and environmental factors for a very limited number of species. Generally speaking, well-known species groups are implicitly used as an indicator group for other species. We found that three of four test analyses of the indicative performance of a species groups proved to perform well, while one indicator species group indicated the species of the higher taxonomic group poorly. The matching importance of some attributes between taxonomic groups shows that these attributes are of key importance and may help to focus conservation policy. We should emphasize, however, that this does not necessarily mean that the different taxonomic groups will show identical responses to conservation measurements based on these attributes. Conservation measures for butterflies may not be effective for other insects, though. Given that insects are by far the most species-rich class of eukaryotes, this is a conclusion of great concern.

Our study shows that it is possible to construct models based on limited taxonomic groups that predict threats to all species based on their traits and requirements. More importantly, it is possible to check the indicative value of species groups, provided sufficient information is available on the Red List status of species from different groups. As this type of information is becoming increasingly available, such checks should become standard procedure.

## Supporting Information

**Table S1.** Dutch red lists used in this study. N spec.: number of Dutch indigenous and reproducing species; Eval.: number of species evaluated for the Red List; Sel.: number of species selected for this study. Number of Dutch species is based on the most recent list of (Noordijk et al. 2010).

**Table S2.** List of attributes. Availability: Ev, evaluated species; We, well-known species; Po, poorly-known species. Species group: An, animals; Pl, plants; Mu, mushrooms.

**Figure S1.** Definitions of prevalence, correct classification, Type I error and Type II error probability.

**Figure S2.** Theoretical effect of prevalence, that is, the number of declining species divided by the total number of species, of the species group and Type I and Type II error probabilities on correct classifications. The effect of three examples of combinations of Type I and Type II error probabilities are shown.
