# Supplementary material for: Predicting rarity and decline in animals, plants, and mushrooms based on species attributes and indicator groups
Source: Ecol Evol. 2013 Aug 28;3(10):3401–14. doi: 10.1002/ece3.699 (PMC3797487; doi:10.1002/ece3.699)
Supplement: Supplementary file 1 [file ece30003-3401-SD1.docx]

**Supporting Information**

**Materials and methods**

**Correcting classification scores for difference in prevalence**

Comparing the classification of the indicator group by its random forest with the classification of the higher taxonomic group by the same indicator random forest is not straightforward. This is because the prevalence of the indicator group, i.e., the proportion of the species that is actually decreasing (Fig. S1), will be different from that of the higher taxonomic group. When the indicator random forest is able to classify the species of the higher taxonomic group with the same accuracy as the species of the indicator group, the probability of type I and type II errors in classifying the two groups is equal. In that case, the prevalence can be used to calculate the expected correct classification of the higher taxonomic group as follows (symbols are according to Fig. S1):

The number of decreasing species in the higher taxonomic group is p_h_*S_h_, where p_h_ is the prevalence of the higher taxonomic group and S_h_ the number of species in the higher taxonomic group. The expected number of correctly classified decreasing species is then (1-II_i_)*p_h_*S_h_, where II_i_ is the probability of type II errors in the indicator random forest. The number of non-decreasing species is (1-p_h_)*S_h_ and the expected number of correctly classified non-decreasing species is (1-I_i_)*(1-p_h_)*S_h_. So the expected total number of correctly classified species is (1-II_i_)*p_h_*S_h_ + (1-I_i_)*(1-p_h_)*S_h_. This gives:

Expected c_h_ = ((1-II_i_)*p_h_*S_h_ + (1-I_i_)*(1-p_i_)*S_h_)/S_h_ = (1-II_i_)*p_h_ + (1-I_i_)*(1-p_h_) (S1)

Fig. S2 shows that the relationship between prevalence p and correct classification c depends on the difference between type I and type II error probabilities. Only when type I and type II errors have the same probability will the expected correct classification of the higher taxonomic group be consistently equal to the correct classification of the indicator group.

**References used for table 2**

Bekker, R.M. & Kwak, M.M. (2005) Life history traits as predictors of plant rarity with particular reference to hemiparasitic *Orobanchaceae*. *Folia Geobotanica*, **40**, 231-242.

Collen, B., Bykova, E., Ling, S., Milner-Gulland, E.J. & Purvis, A. (2006) Extinction risk: a comparative analysis of Central Asian vertebrates. *Biodiversity and Conservation*, **15**, 1859-1871.

Davies, K.F., Margules, C.R. & Lawrence, J.F. (2004) A synergistic effect puts rare, specialized species at greater risk of extinction. *Ecology*, **85**, 265-271.

Dennis, R.L.H., Shreeve, T.G. & Dyck, H. van (2006) Habitats and resources: the need for resource-based definition to conserve butterflies. *Biodiversity and Conservation*, **15**, 1943-1966.

Dulvy, N.K. & Reynolds, J.D. (2002) Predicting extinction vulnerability in skates. *Conservation Biology*, **16**, 440-450.

Fréville, H., McConway, K., Dodd, M. & Silvertown, J. (2007) Prediction of extinction in plants: interaction of extrinsic threats and life history traits. *Ecology*, **88**, 2662-2672.

Harcourt, A.H., Coppeto, S.A. & Parks, S.A. (2002) Rarity, specialization and extinction in primates. *Journal of Biogeography*, **29**, 445-456.

Hero, J-M., Williams, S.E. & Magnusson, W.E. (2005) Ecological traits of declining amphibians in upland areas of eastern Australia. *Journal of Zoology*, **267**, 221-232.

Jennings, N. & Pocock, M.J.O. (2009) Relationships between sensitivity to agricultural intensification and ecological traits of insectivorous mammals and arthropods. *Conservation Biology*, **23**, 1195-1203.

Jiguet, F., Gadot, A.S., Julliard, R., Newson, S.E. & Couvet, D. (2007) Climate envelope, life history traits and the resilience of birds facing global change. *Global Change Biology*, **13**, 1672-1684.

Jiguet, F., Gregory, R.D., Devictor, V., Greene, R.E., Vorísek, P., Strien, A. van & Couvet, D. (2010) Population trends of European common birds are predicted by characteristics of their climatic niche. *Global Change Biology*, **16**, 497-505.

Jones, K.E., Purvis, A. & Gittleman, J.L. (2003) Biological correlates of extinction risk in bats. *The American Naturalist*, **161**, 601-614.

Jones, M.J., Fielding, A. & Sullivan, M. (2006) Analysing extinction risk in parrots using decision trees. *Biodiverisy and Conservation*, **15**, 1993-2007.

Julliard, R., Jiguet, F. & Couvet, D. (2003) Common birds facing global changes : what makes a species at risk? *Global Change Biology*, **10**, 148-154.

Keane, A., Brooke, M. de L. & Mcgowan, P.J.K. (2005) Correlates of extinction risk and hunting pressure in gamebirds (Galliformes). *Biological Conservation*, **126**, 216-233.

Kleukers, R. & Reemer, M. (2003) Verandering in de Nederlandse ongewerveldenfauna. *De Levende Natuur*, **104**, 86-89.

Kooyman, R. & Rossetto, M. (2008) Definition of plant functional groups for informing implementation scenarios in resource-limited multi-species recovery planning. *Biodiversity Conservation*, **17**, 2917-2937.

Kotiaho, J.S., Kaitala, V., Komonen, A. & Päivinen, J. (2005) Predicting the risk of extinction from shared ecological characteristics. *Proceedings of the National Academy of Science*, **102**, 1963-1967.

Lockwood, J.L., Russell, G., Gittleman, J.L., Daehler, C.C., McKinney, M.L. & Purvis, A. (2002) A metric for analyzing taxonomic patterns of extinction risk. *Conservation Biology*, **16**, 1137-1142.

Mattila, N., Kaitala, V., Komonen, A., Kotialho, J.S. & Päivinen, J. (2006) Ecological determinants of distribution decline and risk of extinction in moths. *Conservation Biology*, **20**, 1161-1168.

Mattila, N., Kotiaho, J.S., Kaitala, V. & Komonen, A. (2008) The use of ecological traits in extinction risk assessments: A case study on geometrid moths. *Biological Conservation*, **141**, 2322-2328.

Murray, B.R., Thrall, P.H., Gill, A.M. & Nicotra, A.B. (2002) How plant life-history and ecological traits relate to species rarity and commonness at varying spatial scales. *Austalian Ecology*, **27**, 291-310.

Olden, J.D., LeRoy Poff, N. & Bestgen, K.R. (2008) Trait synergisms and the rarity, extirpation, and extinction risk of desert fishes. *Ecology*, **89**, 847-856.

Purvis, A., Gittleman, J.L., Cowlishaw, G. & Mace, G.M. (2000) Predicting extinction risk in declining species. *Proceedings of the Royal Society B*, **267**, 1947-1952.

Rubach, M. (2010) *Predicting the response of aquatic invertebrates to stress using species traits and stressor mode of action.* PhD thesis, Wageningen University.

Scholes, R.J, Pickett, G., Ellery, W.N. & Blackmore, A.C. (1997) Plant functional types in African savannes and grasslands. *Plant Functional Types. Their relevance to ecosystem properties and global change* (eds T.M. Smith, H.H. Shugart & F.I. Woodward), pp. 255-268. Cambridge University Press, Cambridge.

Sullivan, M.S., Jones, M.J., Lee, D.C., Marsden, S.J., Fielding, A.H. & Young, E.V. (2006) A comparison of predictive methods in extinction risk studies: contrasts and decision trees. *Biodiversity and Conservation*, **15**, 1977-1991.

Turnhout, C.A.M. van, Foppen, R.P.B., Leuven, R.S.E.W., Strien, A. van & Siepel, H. (2010) Life-history and ecological correlates of population change in Dutch breeding birds. *Biological Conservation*, **143**, 173-181.

Vandewalle, M., Bello, F. de, Berg, M.P., Bolger, T., Doléde, S., Dubs, F., Feld, C.K., Harrington, R., Harrison, P.A., Lavorel, S., Martins da Silva, P., Moretti, M., Niemelä, J., Santos, P., Sattler, T., Sousa, J.P., Sykes, M.T., Vanbergen, A.J. & Woodcock, B.A. (2010) Functional traits as indicators of biodiversity response to land use changes across ecosystems and organisms. *Biodiversity and Conservation*, **19**, 2921-2947.

Verberk, W.C.E.P., 2008. *Matching species to a changing landscape. Aquatic macroinvertebrates in a heterogeneous landscape.* PhD thesis, Radboud University Nijmegen.

Walker, K.J. & Preston, C.D. (2006) Ecological predictors of extinction risk in the flora of lowland England, UK. *Biodiversity and Conservation*, **15**, 1913-1942.

Williams, S.E., Shoo, L.P., Isaac, J.L., Hoffmann, A.A. & Langham, G. (2008) Towards an integrated framework for assessing the vulnerability of species to climate change. *PLoS Biology,* **6**, 2612-2626.

**References**

Aptroot, A., Herk, C.M. van, Dobben, H.F. van, Boom, P.P.G. van den, Brand, A.M. & Spier, L. (1998) Bedreigde en kwetsbare korstmossen in Nederland. Basisrapport met voorstel voor de Rode Lijst. *Buxbaumiella*, **46**, 1-101.

Arnolds, E. & Veerkamp, M. (2008) *Basisrapport Rode Lijst paddenstoelen*. Nederlandse Mycologische Vereniging, Utrecht.

Bruyne, R.H. de, Wallbrink, H. & Gmelig Meyling, A.W. (2003) *Bedreigde en verdwenen land- en zoetwatermollusken in Nederland (Mollusca). Basisrapport met voorstel voor de Rode Lijst*. European Invertebrate Survey Nederland, Leiden & Stichting Anemoon, Heemstede.

Delft, J.J.C.W. van, Creemers, R.C.M. & Spitzen-van der Sluijs, A. (2007) *Basisrapport Rode Lijsten amfibieën en reptielen volgens Nederlandse en IUCN-criteria*. Stichting RAVON, Nijmegen.

Hustings F., Borggreve, C., Turnhout, C. van & Thissen, J. (2004) *Basisrapport voor de Rode Lijst vogels volgens Nederlandse en IUCN-criteria*. SOVON Vogelonderzoek Nederland, Beek-Ubbergen.

Meijden, R. van der, Odé, B., Groen, C.L.G., Witte, F.J. & Bal, D. (2000) Bedreigde en kwetsbare vaatplanten in Nederland. Basisrapport met voorstel voor de Rode Lijst. *Gorteria*, **26**, 85-208.

Nie, H.W. de & Ommering, G. van (1998) *Bedreigde en kwetsbare zoetwatervissen in Nederland: Toelichting op de Rode Lijst*. IKC Natuurbeheer, Wageningen.

Noordijk, J., Kleukers, R.M.J.C., Nieuwkerken, E.J. van & Loon, A.J. van (Eds) (2010) *De Nederlandse biodiversiteit.* Nederlandse Fauna 10, Nederlands Centrum voor Biodiversiteit Naturalis & European Invertebrate Survey, Leiden.

Odé, B. (1999) *Bedreigde en kwetsbare sprinkhanen en krekels (Orthoptera). Basisrapport met voorstel voor de Rode Lijst*. European Invertebrate Survey Nederland, Leiden.

Peeters, T.M.J. & Reemer, M. (2003) *Bedreigde en verdwenen bijen in Nederland (Apidae s.l.). Basisrapport met voorstel voor de Rode Lijst*. European Invertebrate Survey Nederland, Leiden.

Siebel, H.N. & Bijlsma, R.J. (2004) Bedreigde en kwetsbare mossen in Nederland: Correcties op het Basisrapport (Buxbaumiella 54). *Buxbaumiella*, **68**, 56-64.

Siebel, H.N., Tooren, B.F. van, Melick, H.M.H. van, Bouman, A.C., During, H.J. & Dort, K.W. van (2000) Bedreigde en kwetsbare mossen in Nederland. Basisrapport met voorstel voor de Rode Lijst. *Buxbaumiella*, **54**.

Swaay, C.A.M. van (2006) *Basisrapport Rode Lijst dagvlinders*. De Vlinderstichting, Wageningen.

Verdonschot, P.F.M., Higler, B.W.G., Nijboer, R.C. & Hoek, T.H. van den (2003) *Naar een doelsoortenlijst van aquatische macrofauna in Nederland; platwormen (Tricladida), steenvliegen (Plecoptera), haften (Ephemeroptera) en kokerjuffers (Trichoptera)*. Alterra, Wageningen.

Wasscher, M., Keijl, G.O. & Ommering, G. van (1998) *Bedreigde en kwetsbare libellen in Nederland*. IKC Natuurbeheer, Wageningen.

Zoogdiervereniging VZZ (2007) *Basisrapport voor de Rode Lijst zoogdieren volgens Nederlandse en IUCN-criteria*, 2nd edn. Zoogdiervereniging VZZ, Arnhem.

**Tables**

**Table S1.** Dutch red lists used in this study. N spec.: number of Dutch indigenous and reproducing species; Eval.: number of species evaluated for the Red List; Sel.: number of species selected for this study. Number of Dutch species is based on the most recent list of Noordijk et al. 2010.

| **Group** | **N spec.** | **Eval.** | **Sel.** | **Published by** |
| --- | --- | --- | --- | --- |
| **Animals** |  |  |  |  |
| Reptiles | 7 | 7 | 7 | Van Delft, Creemers & Spitzen-van der Sluijs 2007 |
| Amphibians | 16 | 16 | 16 | Van Delft, Creemers & Spitzen-van der Sluijs 2007 |
| Planarian, Tricladida | 18 | 11 | 11 | Verdonschot *et al.* 2003 |
| Stoneflies | 27 | 20 | 20 | Verdonschot *et al.* 2003 |
| Grasshoppers | 46 | 43 | 35 | Odé 1999 |
| non-marine fishes | ±50 | 44 | 36 | De Nie & van Ommering 1998 |
| Mayflies | 57 | 52 | 41 | Verdonschot *et al.* 2003 |
| non-marine mammals | 60 | 52 | 41 | Zoogdiervereniging VZZ 2007 |
| Dragonflies | 65 | 61 | 47 | Wasscher, Keijl & van Ommering 1998 |
| Butterflies | 78 | 71 | 50 | Van Swaay 2006 |
| non-marine Mollusks | 166 | 147 | 65 | De Bruyne, Wallbrink & Gmelig Meyling 2003 |
| Caddisflies | 180 | 155 | 69 | Verdonschot et al. 2003 |
| Birds | 186 | 178 | 77 | Hustings *et al.* 2004 |
| Bees | 350 | 322 | 116 | Peeters & Reemer 2003 |
| **Plants** |  |  |  |  |
| Lichenes | 943 | 416 | 80 | Aptroot *et al.* 1998 |
| Bryophytes | 490 | 515 | 86 | Siebel *et al.* 2000; Siebel & Bijlsma 2004 |
| Vascular plants | 1582 | 1582 | 137 | Van der Meijden *et al.* 2000 |
| **Fungi** |  |  |  |  |
| Mushrooms | 4732 | 2405 | 249 | Arnolds & Veerkamp 2008 |

**Table S2.** List of attributes. Availability: Ev: evaluated species; We: well-known species; Po: poorly-known species. Species group: An: animals; Pl: plants; Mu: mushrooms

| Code | | Name |  | Availability | Group | N cat. | Description |
| --- | --- | --- | --- | --- | --- | --- | --- |
| 1 |  | Number of species in genus in Europe |  | Ev We Po | An Pl Mu | 5 | Ln(number) |
| 2 |  | Number of subspecies in Europe |  | Ev We Po | An Pl | 5 | Ln(number) |
| 3 |  | Controlled by man |  | Ev We Po | An | 2 | Y/N |
| 4 |  | Harvested/hunted by man |  | Ev We Po | An Pl | 2 | Y/N |
| 6 |  | Origin |  | Ev We Po | An Pl | 3 | Native, established introduced, newly introduced |
| 10 |  | Body size |  | Ev We Po | An Pl Mu | 4 | <1 cm, 1-10 cm, 11-100 cm, >100 cm |
| 11 | a | Border of range through the Netherlands | Northern | Ev We | An Pl | 2 | Y/N |
|  | b |  | Southern | Ev We | Pl | 2 | Y/N |
|  | c |  | Western | Ev We | An Pl | 2 | Y/N |
| 12 |  | Endemic |  | Ev We Po | An Pl | 3 | Western Europe, Europe, Beyond Europe |
| 13 | a | Shift of border of range 1900-1990 | Northwards | Ev | An Pl | 2 | Y/N |
|  | b |  | Southwards | Ev | An Pl | 2 | Y/N |
|  | c |  | Eastwards | Ev | An Pl | 2 | Y/N |
|  | d |  | Westwards | Ev | An Pl | 2 | Y/N |
|  | e |  | No shift | Ev | An Pl | 2 | Y/N |
| 14 | a | Shift of border of range since 1990 | Northwards | Ev | An Pl Mu | 2 | Y/N |
|  | b |  | Southwards | Ev | An Mu | 2 | Y/N |
|  | c |  | Eastwards | Ev | An Mu | 2 | Y/N |
|  | d |  | Westwards | Ev | An Pl Mu | 2 | Y/N |
|  | e |  | No shift | Ev | An Pl Mu | 2 | Y/N |
|  | n |  | Other | Ev | Pl | 2 | Y/N |
| 15 | a | Main habitat | Marine | Ev We Po | An | 2 | Y/N |
|  | b |  | Fresh water | Ev We Po | An Pl | 2 | Y/N |
| 16 | a | Species found in forest or non-forest habitats | Almost exclusively non-forest | Ev We | An Mu | 2 | Y/N |
|  | b |  | Mainly non-forest | Ev We | An Mu | 2 | Y/N |
|  | c |  | Both forest and non-forest habitats | Ev We | An Mu | 2 | Y/N |
|  | d |  | Mainly forest | Ev We | An Mu | 2 | Y/N |
|  | e |  | Almost exclusively forest | Ev We | An Mu | 2 | Y/N |
| 17 | a | Terrestrial humidity | Dry | Ev We | An Pl Mu | 2 | Y/N |
|  | b |  | Humid | Ev We | An Pl Mu | 2 | Y/N |
|  | c |  | Wet | Ev We | An Pl Mu | 2 | Y/N |
|  | d |  | No preference | Ev We | An Pl | 2 | Y/N |
| 18 | a | Aquatic habitats | Running | Ev We Po | An | 2 | Y/N |
|  | b |  | Stagnant | Ev We Po | An Pl | 2 | Y/N |
|  | c |  | No preference | Ev We Po | An | 2 | Y/N |
| 19 |  | Dependence on natural habitats |  | Ev | An Pl Mu | 5 | Almost exclusively natural, mainly natural, both natural and non-natural (urban/agricultural), mainly non-natural, almost exclusively non-natural |
| 20 | a | Preference for urban or agricultural habitats | Agricultural | Ev | An Pl Mu | 2 | Y/N |
|  | b |  | Infra-structure | Ev | An Pl Mu | 2 | Y/N |
|  | c |  | Urban | Ev | An Pl | 2 | Y/N |
| 21 |  | Preference habitat stability |  | Ev | An Pl Mu | 5 | Only stable, mainly stable, no clear preference, mainly dynamic, only dynamic |
| 22 | a | Reproductive period | April-June | Ev We | An Pl Mu | 2 | Y/N |
|  | b |  | July-September | Ev We | An Pl Mu | 2 | Y/N |
|  | c |  | April-September | Ev We | An Pl | 2 | Y/N |
|  | d |  | October-March | Ev We | An Pl Mu | 2 | Y/N |
|  | n |  | Other | Ev We | Mu | 2 | Y/N |
| 23 | a | Food, non-adults | Photosynthesis | Ev We Po | An Mu | 2 | Y/N |
|  | b |  | Dead organic material | Ev We Po | An Mu | 2 | Y/N |
|  | c |  | Parasite | Ev We Po | An Mu | 2 | Y/N |
|  | d |  | Predator | Ev We Po | An | 2 | Y/N |
|  | e |  | Herbivore | Ev We Po | An | 2 | Y/N |
|  | f |  | Omnivore | Ev We Po | An | 2 | Y/N |
| 24 | a | Food, adults | Photosynthesis | Ev We Po | An Mu | 2 | Y/N |
|  | b |  | Dead organic material | Ev We Po | An Mu | 2 | Y/N |
|  | c |  | Parasite | Ev We Po | An | 2 | Y/N |
|  | d |  | Predator | Ev We Po | An | 2 | Y/N |
|  | e |  | Herbivore | Ev We Po | An | 2 | Y/N |
|  | f |  | Omnivore | Ev We Po | An | 2 | Y/N |
|  | g |  | Not eating | Ev We Po | An | 2 | Y/N |
| 25 |  | Dispersion capacity |  | Ev We | An Pl Mu | 4 | <100 m, 100 m-1 km, 1-10 km, >10 km |
| 26 |  | Considered a pest |  | Ev We Po | An | 2 | Y/N |
| 27 |  | More than 50% of distribution in agricultural area before 1960 | | Ev | An Pl | 2 | Y/N |
| 28 |  | More than 50% of distribution in agricultural area after 1960 | | Ev | An Pl | 2 | Y/N |
| 29 |  | Number of generations per year |  | Ev We | An Pl Mu | 3 | Less than one, one, more than one |
| 30 |  | Non-adult stages morphologically different | | Ev We Po | An Pl | 2 | Y/N |
| 31 |  | Years before reproduction |  | Ev We Po | An Pl | 2 | One year, two or more years |
| 32 |  | Reproductive years |  | Ev We Po | An Pl | 3 | One, two to five, more than five |
| 33 | a | Reproduction | Sexual | Ev We Po | An Pl | 2 | Y/N |
|  | b |  | Non-sexual | Ev We Po | An Pl | 2 | Y/N |
|  | c |  | Both | Ev We Po | Pl | 2 | Y/N |
| 34 |  | Depending on symbioses |  | Ev We Po | An Pl Mu | 2 | Y/N |
| 35 | a | Marine substrate | Solid | Ev We Po | An | 2 | Y/N |
|  | b |  | Non-solid | Ev We Po | An | 2 | Y/N |
| 36 |  | Reproductive area |  | Ev We Po | An | 4 | <100 m^2^, < 1 hectare, < 1 km^2^, >1 km^2^ |
| 37 | a | Endotherm |  | Ev We Po | An | 2 | Y/N |
| 38 | a | First winter as | Egg | Ev We | An | 2 | Y/N |
|  | b |  | Larvae | Ev We | An | 2 | Y/N |
|  | c |  | Egg or larvae | Ev We | An | 2 | Y/N |
|  | d |  | Nymph | Ev We | An | 2 | Y/N |
|  | e |  | Subadult or adult | Ev We | An | 2 | Y/N |
|  | f |  | Different stages | Ev We | An | 2 | Y/N |
| 39 | a | Adult activity | Night | Ev We | An | 2 | Y/N |
|  | b |  | Day | Ev We | An | 2 | Y/N |
| 40 | a | Herbivore diet | Vascular herbs | Ev We | An | 2 | Y/N |
|  | b |  | Vascular bushes and trees | Ev We | An | 2 | Y/N |
|  | c |  | Non-vascular plants | Ev We | An | 2 | Y/N |
|  | d |  | Different plants | Ev We | An | 2 | Y/N |
| 41 | a | Herbivores depending on | One species | Ev We | An | 2 | Y/N |
|  | b |  | One genus | Ev We | An | 2 | Y/N |
|  | c |  | Limited number of genera | Ev We | An | 2 | Y/N |
|  | d |  | Not depending on limited species | Ev We | An | 2 | Y/N |
|  | e |  | Unknown | Ev We | An | 2 | Y/N |
| 42 | a | Predators depending on | One species | Ev We | An | 2 | Y/N |
|  | b |  | Limited number of species | Ev We | An | 2 | Y/N |
|  | c |  | Not depending on limited species | Ev We | An | 2 | Y/N |
| 43 | a | Parasites depending on | One species | Ev We | An | 2 | Y/N |
|  | b |  | Limited number of species | Ev We | An | 2 | Y/N |
|  | c |  | Not depending on limited species | Ev We | An | 2 | Y/N |
| 44 | a | Social animals | Yes | Ev We | An | 2 | Y/N |
|  | b |  | No | Ev We | An | 2 | Y/N |
| 45 | a | Flying | Flying stage present | Ev We Po | An | 2 | Y/N |
|  | b |  | Flying stage absent | Ev We Po | An | 2 | Y/N |
| 46 |  | Active dispersion |  | Ev We | An | 4 | Without wings, with wings but not outside reproductive area, with wings often outside reproductive area, migrating species |
| 47 | a | Passive dispersion | By man | Ev We | An | 2 | Y/N |
|  | b |  | By animals | Ev We | An | 2 | Y/N |
|  | d |  | No passive dispersion | Ev We | An | 2 | Y/N |
| 48 |  | Number of offspring per year |  | Ev We Po | An | 5 | 1-3, 4-10, 10-100, 100-1000, >1000 |
| 49 | a | Habitat non-adult | Different from adults | Ev We | An | 2 | Y/N |
|  | b |  | Not different | Ev We | An | 2 | Y/N |
| 50 | a | Growing habitat | Shaded | Ev We Po | Pl Mu | 2 | Y/N |
|  | b |  | Non-shaded | Ev We Po | Pl Mu | 2 | Y/N |
|  | c |  | No preference | Ev We Po | Pl Mu | 2 | Y/N |
| 51 |  | Functional group |  | Ev We Po | Pl | 10 | Raunkiaer classification |
| 52 | b | Pollination | Wind | Ev We Po | Pl | 2 | Y/N |
|  | c |  | Insects | Ev We Po | Pl | 2 | Y/N |
| 53 | a | Dispersion of seeds | Wind | Ev We Po | Pl Mu | 2 | Y/N |
|  | b |  | Water | Ev We Po | Pl | 2 | Y/N |
|  | c |  | Animals | Ev We Po | Pl Mu | 2 | Y/N |
|  | d |  | Gravity | Ev We Po | Pl Mu | 2 | Y/N |
| 54 | a | Seed longevity | 1-3 years | Ev We Po | Pl | 2 | Y/N |
|  | b |  | 4-10 years | Ev We Po | Pl | 2 | Y/N |
|  | c |  | More than 10 years | Ev We Po | Pl | 2 | Y/N |
| 55 | a | Winter leaf carrying |  | Ev We Po | Pl | 2 | Y/N |
| 56 | a | Land use category | Coniferous or mixed woods | Ev | An Pl Mu | 5 | Phi |
|  | b |  | Deciduous woods | Ev | An Pl Mu | 5 | Phi |
|  | c |  | Grassland | Ev | An Pl Mu | 5 | Phi |
|  | d |  | Arable land | Ev | An Pl Mu | 5 | Phi |
|  | e |  | Orchards | Ev | An Pl Mu | 5 | Phi |
|  | f |  | Wetlands | Ev | An Pl Mu | 5 | Phi |
|  | g |  | Heathland and bogs | Ev | An Pl Mu | 5 | Phi |
|  | h |  | Dunes and bare sands | Ev | An Pl Mu | 5 | Phi |
|  | i |  | Open water | Ev | An Pl Mu | 5 | Phi |
|  | j |  | Estuarine marshland and tidal sand plates | Ev | An Pl Mu | 5 | Phi |
|  | k |  | Fens | Ev | An Pl Mu | 5 | Phi |
|  | l |  | Urban green | Ev | An Pl Mu | 5 | Phi |
|  | m |  | Urban area | Ev | An Pl Mu | 5 | Phi |
|  | s |  | Specialization | Ev | An Pl Mu | 5 | Sqr SS |
| 57 | a | Physical-geographical region | River clay | Ev | An Pl Mu | 5 | Phi |
|  | b |  | Peat | Ev | An Pl Mu | 5 | Phi |
|  | c |  | Marine clay | Ev | An Pl Mu | 5 | Phi |
|  | d |  | Dune and sea sand | Ev | An Pl Mu | 5 | Phi |
|  | e |  | Pleistocene sand | Ev | An Pl Mu | 5 | Phi |
|  | f |  | Loess | Ev | An Pl Mu | 5 | Phi |
|  | g |  | Old clay | Ev | An Pl Mu | 5 | Phi |
|  | h |  | Anthropogenic | Ev | An Pl Mu | 5 | Phi |
|  | i |  | Open water | Ev | An Pl Mu | 5 | Phi |
|  | s |  | Specialization | Ev | An Pl Mu | 5 | Sqr SS |
| 59 |  | Commonness 1950-90 |  | Ev | An Pl Mu | 5 | Logit(number of grid cells species/number of grid cells species group) |
| 61 |  | Nutrient indication |  | Ev We | Pl | 9 | Ellenberg indication value |
| 62 |  | pH indication |  | Ev We | Pl | 9 | Ellenberg indication value |
| 63 |  | Functional groups mushrooms |  | Ev We Po | Mu | 8 | Ectomycorrhiza; ectendomycorrhiza; ericoid mycorrhiza; associated with mosses biotrophic parasite; necrotrophic parasite; saprotroof (dung); saprotroof (wood); saprotroof (herbs); saprotroof (soil organic matter). |
| 64 |  | Taxonomic groups mushrooms |  | Ev We Po | Mu | 5 | Agaricales, Aphyllophorales, Ascomycotina, Gasteromycetes, Phragmobasidiomycetidae |
| 65 |  | Sensitivity to eutrophication |  | Ev | Mu | 3 | Negative, no effects, possitive |

**Figures legends**

**Fig. S1.** Definitions of prevalence, correct classification, type I error and type II error probability

**Fig. S2.** Theoretical effect of prevalence, i.e., the number of declining species divided by the total number of species, of the species group and type I and type II error probabilities on correct classifications. The effect of three examples of combinations of type I and type II error probabilities are shown.

**Figures**

Figure S1

| Decline | | Classification | |
| --- | --- | --- | --- |
|  |  | No | Yes |
| Declining | No | TN | FP |
|  | Yes | FN | TP |

T: true

F: false

N: negative

P: positive

Prevalence: p = (FN+TP)/(TN+FP+FN+FP)

Correct classification: c = (TN+TP)/(TN+FP+FN+TP)

Type I error rate: I = FP/(TN+FP)

Type II error rate: II = FN/(FN+TP)

All species: S = TN+FP+FN+TP

Figure S2
